# Bike racing, recreational riding, impact sport and bone health

**DOI:** 10.1186/1741-7015-10-169

**Published:** 2012-12-20

**Authors:** Michael R Carmont

**Affiliations:** 1 Princess Royal Hospital, Shrewsbury and Telford NHS Trust, Telford and the Northern General Hospital, Sheffield Teaching Hospitals NHS Foundation Trust, Sheffield, UK

**Keywords:** cycling, mountain biking, osteoporosis, anterior cruciate ligament

## Abstract

Cycling has been shown to confer considerable benefits in terms of health, leading to reductions in death rates principally due to cardiovascular improvements and adaptation.

Given the disparity between the benefits of cycling on cardiovascular fitness and previous research finding that cycling may not be beneficial for bone health, Hugo Olmedillas and colleagues performed a systematic review of the literature. They concluded that road cycling does not appear to confer any significant osteogenic benefit. They postulate that the cause of this is that, particularly at a competitive level, riders spend long periods of time in a weight-supported position on the bike.

Training programs may be supplemented with impact loading to preserve bone health; however, the small increased risk of soft tissue injury must also be considered.

See related commentary http://www.biomedcentral.com/1741-7015/10/168

## Introduction

Cycling has been shown to confer considerable benefits in terms of health. The replacement of car trips with cycling and public transport use in Barcelona has led to a reduction in overall deaths of 66.12 although there were an estimated increase in 1.15 deaths due to air pollution and an additional 0.17 deaths due to road traffic fatalities [1]. Notably the shift led to a reduction of CO_2 _203,251 t/CO_2 _emissions per year. Similar results were seen in the form of a reduction of 19.5 Disability Adjusted Life Years in Copenhagen after the introduction of cycling to the place of work or education [2]. These reductions in death rates are principally due to cardiovascular benefits.

In regard to health benefits, other body systems should be considered. Peak bone density forms in the second and third decades and declines thereafter. Low bone mineral density increases the risk of stress and fragility fractures as a result of the declining bone density typically occuring in later life. Fragility fractures occur when the ultimate tensile strength of bone is exceeded by the forces it is subjected to during activities of daily living as well as the higher forces during falls. It is, therefore, beneficial to optimize peak bone density during adolescence and early adult life during bone formation. Bone will be optimally formed with impact loading according to Wolff's law: that bone tissue forms and is remodelled in response to the mechanical forces that it experiences [3].

When considering bone health, however, the impact of cycling is less beneficial than other sports. Cycling may be considered to be a non-impact sport with reduced weight bearing, and as a result may be expected to lead to comparatively reduced bone density. Given the disparity between the benefits of cycling on cardiovascular fitness and some previous research findings that cycling may not be beneficial for bone health, Hugo Olmedillas and colleagues performed a systematic review of the literature [4].

### Sports and bone health: what are the effects?

This systematic review included 31 studies that analyzed bone mass and bone metabolism in cyclists across four databases. The authors concluded that road cycling does not appear to confer any significant osteogenic benefit. They postulate that the cause of this is that, particularly at a competitive level, riders spend long periods of time in a weight-supported position on the bike. It is hypothesized that this, in combination with a necessary enforced recovery time involving a large amount of time sitting or lying supine, results in low bone mineral density due to a lack of impact [4].

How do the findings of this study influence bone health in cyclists? The clinical implications may not be as clear as one would imagine. For instance, the vast majority of cyclists do not compete at a competitive level and off-road cycling may be more popular in the age group during which peak bone mass is optimized. The choice of bike may also influence bone health. Suspension systems are currently fashionable with bikes being classified as rigid, hard tail or front suspension. With increased suspension, muscular stress has been noted to be increased [5], although no studies have been undertaken regarding bone stress.

Weight bearing exercise is known to be beneficial for long-term bone health and adolescents and adults participating in endurance sports have been shown to have a lower bone mineral density than those participating in ball and power sports [6]. High loading sports such as gymnastics, hurdling, judo, karate and other jumping sports lead to higher bone mineral composition, bone mineral density and enhanced bone geometry specifically related to the sports participated in. Football, basketball, racquet games and step aerobics are described as having odd impact loading and swimming and cycling as non-impact sports [7]. Similarly, collegiate gymnasts have been shown to have significantly higher bone mineral density (BMD) compared to cross country runners [8].

Although beneficial for bone health, high and odd impact sports have been associated with increased risk of anterior cruciate ligament injury. Team handball played by both sexes has one of the highest injury rates, with as many as 2.29 anterior cruciate ligament (ACL) injuries per 1,000 match hours in Norwegian female elite competition [9]. Injury rates in the at-risk population aged 16 to 39 years are as much as 85 per 100,000 and occur in females with a frequency of four times that of males [10]. While reconstructive surgery may allow participants to return to sports activity, this does not occur in every case [11]. Thus, young females may be encouraged to return to non-pivoting, low impact sports, such as swimming and cycling. It is also worthy of note that following ACL injury, the bone mineral density declines in the injured leg and does not return to that of the non-injured leg even after muscular strength returns to normal [12,13].

## Conclusions

Competitive endurance cycling may be considered to have benefits for health but meta-analyses confirm that this does not extend to bone health. Training programs may be supplemented with impact loading to preserve bone health; however, the increased risk of ligamentous injury must be considered with the participation in pivoting sports.

## Abbreviations

ACL: anterior cruciate ligament; BMD: bone mineral density.

## Competing interests

The author declares that they have no competing interests.

## Authors' information

MC is a Consultant Trauma and Orthopaedic Surgeon with a specialist interest in Sports Injuries and Sports Medicine. He is also President of the British Orthopaedic Sports Trauma and Arthroscopy Association.

## Pre-publication history

The pre-publication history for this paper can be accessed here:

http://www.biomedcentral.com/1741-7015/10/169/prepub
